# Pathways and challenges in the clinical translational of radiopharmaceuticals for pediatric investigations

**DOI:** 10.3389/fmed.2025.1658588

**Published:** 2025-11-07

**Authors:** Erik Stauff, Hanieh Karimi, Heidi H. Kecskemethy, Thomas H. Shaffer, Reza Vali, Lauren W. Averill, Xuyi Yue

**Affiliations:** 1 Department of Radiology, Nemours Children’s Health, Wilmington, DE, United States; 2 Diagnostic and Research PET/MR Center, Nemours Children’s Health, Wilmington, DE, United States; 3 Nemours Biomedical Research, Nemours Children’s Health, Delaware, Wilmington, DE, United States; 4 Department of diagnostic imaging and interventional radiology, The Hospital for Sick Children, University of Toronto, Toronto, ON, Canada; 5 Department of Radiology, Thomas Jefferson University, Philadelphia, PA, United States; 6 Department of Psychological and Brain Sciences, University of Delaware, Newark, DE, United States

**Keywords:** pediatric radiopharmaceuticals, FDA guidelines, regulatory pathways, molecular imaging, clinical trial

## Abstract

Radiopharmaceutical development and clinical translation face numerous scientific, ethical, and regulatory challenges, particularly within the pediatric population. Although molecular imaging holds significant promise for improving diagnosis and treatment across a spectrum of diseases, including pediatric-specific conditions like Kawasaki disease, autism spectrum disorders, attention-deficit/hyperactivity disorder, and neuroblastoma, the path from discovery to clinical application remains problematic. The U.S. Food and Drug Administration (FDA) provides three primary pathways—traditional Investigational New Drug (IND) applications, exploratory Investigational New Drug application (eIND), and the Radioactive Drug Research Committee (RDRC) mechanism—to facilitate clinical translation of radiotracers. However, these frameworks are not specifically tailored to pediatric needs. Children’s heightened sensitivity to ionizing radiation, coupled with physiological variability and ethical concerns, complicates trial design, dosimetry, and informed consent. Current practices also exhibit the limitation of inconsistent dosing standards across institutions. Emerging technologies—including improved single-photon emission computed tomography and positron emission tomography techniques, theranostics, whole-body scanners, and artificial intelligence-driven radiomics—offer potential to reduce these risks by enabling lower doses, reduced scan time, and more precise targeting. Nonetheless, a significant gap remains in translating these innovations into safe, equitable access for pediatric patients. Addressing these challenges requires updated regulatory guidance, ethical frameworks, and robust clinical strategies to ensure equitable access to molecular imaging innovations for children.

## Highlights

- Current regulatory pathways for the clinical translation of radiopharmaceuticals are exclusively designed for the adult population.
- A significant gap exists in the investigation of radiopharmaceuticals in pediatric populations due to concerns regarding safety, beneficence, and justice.
- Emerging technologies, such as novel imaging agents, advances in instrument sensitivity, and artificial intelligence-based radiomics, may mitigate some of these risks as they become more established.
- Inter-institutional and international collaborations, as well as data sharing, are crucial for expediting the translation of radiopharmaceuticals for clinical investigation in children.
- Harmonization of regulatory requirements for clinical translation enhances the ethical justification for pediatric trials and facilitates data-driven decision-making.

## Introduction

One of the greatest challenges in medical research is translating scientific discoveries into clinical investigation. How can the science we uncover be transformed into real-world benefit for patients? In drug development, this process is complicated by scientific, ethical, and regulatory barriers. Radiopharmaceutical drug discovery is plagued by all these challenges and additionally bears the burden of ensuring that radiation exposure is medically justified for both treatment and diagnosis purposes. These complexities are further amplified when applied to pediatric medicine. Children have unique physiological and psychological characteristics that necessitate heightened care, more stringent ethical oversight, and specialized consent processes. In the United States (US), several pathways exist for the clinical translation of radiotracers, but none are especially tailored to the needs of pediatric populations. Molecular imaging with radiotracers holds tremendous promises for improving disease management, diagnosis, and treatment, especially in oncology, neurological disorders, and cardiovascular diseases. To unlock this potential, new tracers need to be developed and demonstrated to be safe and effective. However, the development and validation processes are often protracted, resource-intensive, and costly. While rigorous preclinical testing is essential to ensure safety and efficacy, advancing to human trials is required for these agents to reach clinical use. Some diseases, such as Kawasaki disease and neuroblastoma (1–3), are present uniquely in pediatric patients, creating both a need and an opportunity for targeted radiopharmaceutical development. Other diseases, such as gliomas and sarcomas, have high prevalence in the pediatric population, making these pathologies a tempting target for radiotracer development. The up-and-coming tracer fibroblast activation protein inhibitor (FAPI)-based radiotracers have shown higher sensitivity than 2-deoxy-2-[^18^F]fluoro-D-glucose ([^18^F]FDG) for detecting these tumors (4). Overcoming the barriers to clinical translation is crucial. Ultimately, it is imperative to balance the protection of vulnerable pediatric patients with the advancement of scientific and clinical innovation.

## Translational strategies in the United States

Examination of the general pathways of pharmaceutical translation is necessary to better understand the challenges of pediatric radiopharmaceutical translation. In the United States, this process is primarily regulated by the United Sates Food and Drug Administration (FDA). The FDA oversees and approves human trials, while the United States Pharmacopeia (USP) stipulates standards for sterile manufacturing and documentation in drug compounding. Any drug approved for clinical use by the FDA has either a New Drug Application (NDA) or an Abbreviated New Drug Application (ANDA) (5). Radiotracers that have passed through these stages have been evaluated in multiple phases of clinical trials and deemed safe for human use. This includes traditional radiotracers, such as ^99*m*^Tc-based imaging agents and [^18^F]FDG, as well as newer theranostic agents like [^177^Lu]Lu-vipivotide tetraxetan and [^68^Ga]Ga-gozetotide for targeting prostate cancer, and [^68^Ga]Ga-dotatate and [^177^Lu]Lu-dotatate for imaging and treating neuroendocrine tumors, respectively (6). For all other drugs, including radiotracers, the FDA generally provides three primary regulatory pathways for human clinical trials, which generally apply to adult clinical translation. While the research pathway of radiopharmaceuticals shares similarities with the approval process of other drugs, there are important distinctions unique to radiopharmaceuticals that must be understood.

The first pathway involves submitting an Investigational New Drug (IND) application to the FDA. This is the standard way that new radiopharmaceuticals enter the US market and is often associated with a phase 1 clinical trial, or traditional INDs. To initiate such a trial, approval must be obtained from both the FDA (7), and the Institutional Review Board (IRB). The hallmark of an IND is its applicability for all population types. While all populations can technically be included, stage 1 radiopharmaceutical trials are almost exclusively performed on adults. The IND pathway is typically designed for clinical investigation of a single radiopharmaceutical or multiple candidate radiopharmaceuticals with the same chemical class. The primary goal is to determine safety and efficacy. Unlike other approval pathways, which may limit the scope of research, the IND route specifically delves into therapeutic, diagnostic, or preventive use (8). There is no restriction on the number of subjects enrolled in the trial. Due to the comprehensive clinical nature of the IND, a higher burden of proof regarding the drug’s safety is required. The drug must be manufactured under either the current Good Manufacturing Practices (cGMP) as defined by the FDA’s CFR 212 guidelines or under USP 823 standards to ensure proper Chemistry, Manufacturing and Controls (CMC), which are an important part of the IND submission. Robust preclinical documentation must be provided, including dosimetry, toxicology, pharmacologic safety, and genotoxicity data primarily derived from animal studies. While dosimetry is typically performed in rodents, toxicology and pharmacologic safety assessments must also be performed in a second, non-rodent species to demonstrate safety and qualify for a full IND. Once these documents and a detailed clinical protocol are submitted, the FDA reviews the filed documents and evaluates each case individually. After approvals from both the FDA and the IRB are received, clinical trial recruitment may proceed.

The second way that a radiopharmaceutical may be moved to clinical investigation is with an exploratory Investigational New Drug application (eIND), also referred to as a phase 0 clinical trial. While it shares some similarities with the traditional IND in the approval process, the eIND is more limited in both scope and breadth and carries a reduced preclinical burden. Specifically, the eIND is intended only for basic research without a defined therapeutic or diagnostic purpose. Additionally, all eIND studies are limited to microdose level clinical investigation, which are demonstrably sub-therapeutic (9). The microdose level is defined as 100 micrograms (for small molecules) or approximately 1/100th of the no observed adverse effect level. For protein products, the maximum dose is less than or equal to 30 nanomoles (10). The requirements for pharmacologic safety and toxicology are less stringent with an eIND and involve preclinical evaluation in only one species. Rodent dosimetry studies remain necessary, and manufacturing guidelines for radiopharmaceuticals must meet the same standards as those for a traditional IND. However, genotoxicity data are not required at this stage. These characteristics make the eIND pathway particularly suitable for first-in-human trials. Since this type of research occurs between preclinical studies and phase 1 clinical trials, the number of participants is typically less than 30 (8). After this threshold is reached or when the eIND investigation is ended, the eIND must be withdrawn, and the study must be transitioned to a full IND.

**FIGURE 1:**
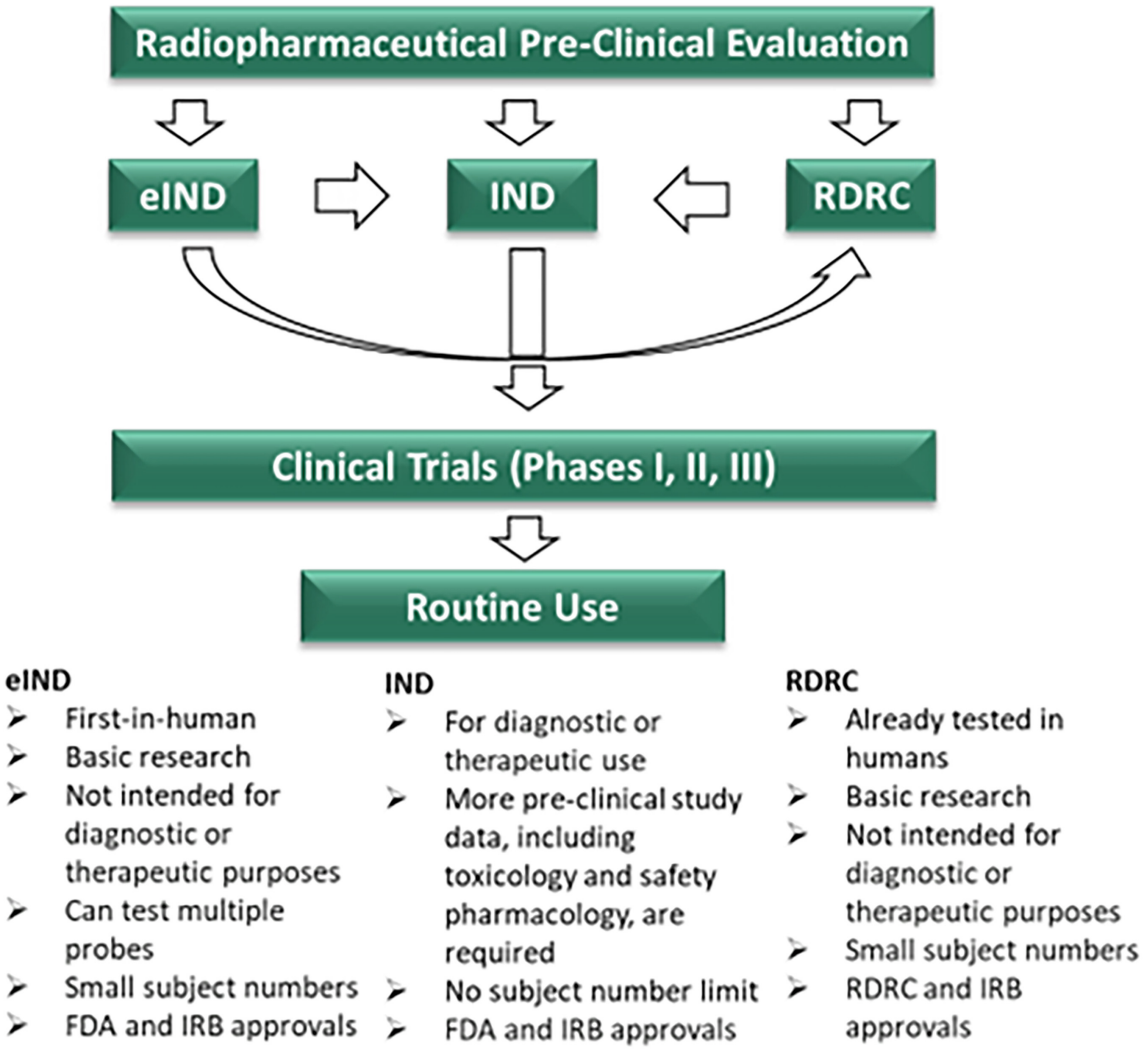
Regulatory pathways for the approval of radiopharmaceuticals in the United States and compassion of eIND, IND, and RDRC mechanisms. eIND, exploratory investigational new drug; IND, investigational new drug; RDRC, Radioactive Drug Research Committee; FDA, the United States food and drug administration; IRB, institutional review board.

The third research pathway for radiopharmaceuticals applies only to radiotracers that have already been approved for human use and utilizes the Radioactive Drug Research Committee (RDRC) program (11). Established under 21 CFR 361, the RDRC program allows basic research using radioactive drugs under specific limitations. Both pharmacological and radiation doses must comply with the thresholds set forth in the regulation. This research may investigate physiology, pathophysiology, or biochemistry, but it cannot assess efficacy, including therapeutic or diagnostic outcomes. Investigators must be properly qualified and licensed to use the approved radiopharmaceutical outside of investigational use. When these conditions are met, an RDRC may be formed to oversee the study. Rather than approving individual studies, the FDA authorizes the committee itself to monitor the research and submit reports. Since this regulatory pathway is applicable only to radiopharmaceuticals that have been tested in human subjects or when the drug is an isotopic substitution of a known agent, it is essentially not suitable for first-in-human trials. However, the RDRC pathway offers advantages, particularly in pediatric research, as it allows the use of radiotracers with established safety profiles. Figure 1 outlines the pathways to bench-to-bedside translation of radiopharmaceuticals in the US and their key differences.

Together, these three regulatory pathways form a framework for achieving FDA approval of clinical trials involving radiopharmaceuticals, but they critically apply primarily to the adult population and are not specifically designed to support pediatric research. Clinical trials for radiopharmaceuticals are almost exclusively conducted in the adult population. It is important to note that the FDA retains a degree of flexibility and does not necessarily enforce a uniform process for any regulatory pathway for clinical investigation. An example is the FDA waiver of IRB approval for emergency use. Sponsors and investigators typically participate in a pre-IND meeting with the FDA, where specific requirements can be clarified or modified based on the unique context of the application. This meeting is a critical step, as it allows preclinical and regulatory elements to be fine-tuned, potentially saving time and resources while ensuring that preclinical research appropriately supports clinical investigation through either mechanism (8).

## Translational strategies in the European Union (EU)

There are complexities with the clinical translation of radiopharmaceuticals in the EU as well. Because the regulations of individual member states (MS) can vary from country to country, efforts have been made to centralize the flow of research information, primarily through Clinical Trial Regulation (CTR) (regulation (EU) No 536/2014). This regulation includes the 27 EU member states as well as the European Economic Area (EEA) states – namely Iceland, Liechtenstein and Norway (12). Similar to how the US regulates IND applications, the EU regulates clinical trials Investigational Medical Products (IMP) through the CTR system. This is accomplished through the Clinical Trials Information System (CTIS), a centralized database that harmonizes clinical oversight between individual member states. This is important because MS/EEA states traditionally had full control over the local clinical trial environment.

Under the current system, each MS/EEA state still holds the responsibility to authorize and monitor clinical trials, but participation in CTIS allows for the flow of information and synergy of research between MS. For each IMP submission, a lead MS submits Part I of the Investigational Medicinal Product Dossier (IMPD), including information on the trial, protocols, risk analysis, and any other pertinent information required by the International Council for Harmonization (ICH) M3 (R2) [European Medicines Agency (13)]. Following this, Part II of the application contains information specific to each Member State’s clinical regulations. Clinical trials must then be approved through each involved MS’s respective competent authority and ethics committees after a single dossier submission through CTIS, after which a unified decision on Part I and Part II is rendered to all the involved member states, with each Member State having authority over Part II. The dossier can be fully approved, approved with conditions, or outright rejected. Individual MS have the authority to reject either part, with a single MS rejection of part I revoking approval (10).

Regarding pediatric trials, the EMA has the Pediatric Investigation Plan (PIP) guidance, which aims to ensure that the necessary data are generated to ensure that products meet stringent standards for authorization in the pediatric population. Ideally, information for the PIP should be submitted upon completion of adult pharmacokinetic data. However, PIP processes can either be waived or converted to a less rigid, stepwise PIP (sPIP) in cases where full PIP processes are not reasonable, such as instances of treating a rare disease or addressing other substantial unmet needs (14).

## Translational strategies in Canada

Canada has a robust system of clinical trial management that also adheres to the same quality standards set forth by GMP and ICH M3, as well as the Canadian Food and Drug Act. In place of an IND or IMP, Health Canada uses a Clinical Trial Application via a New Drug Submission (NDS) for human drug trials. Depending on the nature of the clinical trial, the NDS is overseen by a specific Directorate. In the case of radiopharmaceuticals, which are classified under Schedule C by Division 3 of the Food and Drug Act, the Biologics and Genetic Therapies Directorate (BGTD). The required information includes Module I, which covers safety and efficacy information, and Module II, which covers the quality of chemistry and manufacturing.

Health Canada has a pilot regulatory framework for pediatric studies where sponsors can participate in a Canadian pediatric development plan (C-PDP) or import a PIP from the EU (EU-PIP). This program follows the standards outlined in international guidelines, including ICH S11 and E11, as well as FDA Pediatric Study Plan (PSP) and EU PIP, which specify the proper inclusion of pediatric safety information for pharmaceuticals. Table 1 highlights the similarities between clinical trial initiation in these different countries.

**TABLE 1:** A comparison of three major research jurisdictions: the US, the EU, and Canada.*

Category	US	EU	Canada
Regulatory body	FDA	EMA	Health Canada
Pediatric inclusion	Pediatric studies required if indication includes children; extrapolation allowed with justification	Pediatric use considered in PIP; age-appropriate dosing and safety data required	Pediatric data required if pediatric population is targeted
Toxicity requirements	Non-clinical studies must assess ligand and radiation toxicity; juvenile animal studies case-by-case	Toxicity studies focus on chemical toxicity; radiation toxicity assessed via dosimetry	Toxicity data must support safety; includes ligand and radionuclide toxicity; juvenile studies if needed
Dosimetry requirements	Animal biodistribution and dosimetry required; human dosimetry in Phase 1–3; ALARA principle applied	Radiation dosimetry mandatory: includes biodistribution, decay kinetics, and absorbed dose estimates	Dosimetry required for market authorization includes organ-specific uptake and exposure modeling
Microdose definition	≤ 100 μg or ≤1/100th of active dose; 30 nmol for protein products	Same as FDA [ICH M3 (R2)]	Same as FDA [ICH M3 (R2)]
Exploratory trials allowed	Yes, under exploratory IND	Yes, under ICH M3 (R2)	Yes, under ICH M3 (R2)
Cassette microdosing	Permitted	Permitted	Permitted
Compassionate access program	Expanded access/emergency use	Named patient/compassionate use	Special access program
Initiator of request	Physician or sponsor	Physician	Physician
Major components for clinical trials	CMC, toxicology, dosimetry, clinical protocol, investigator’s brochure, IRB approval	IMPD, clinical protocol, investigator’s brochure including toxicology., dosimetry and pharmacology, ethics board approval	CMC, clinical protocol, investigator’s brochure (including toxicology, dosimetry, and pharmacology), ethics board approval
Approval time	30 days for initial study; 0–30 days for amendments	60 days	30 days

*US, the United States; EU, the European Union; FDA, the United States food and drug administration; EMA, European Medicines Agency; PIP, pediatric investigation plan; ALARA, as low as reasonably achievable; ICH, the International Council for Harmonization; IND, investigational new drug; CMC, chemistry, manufacturing, and controls; IMPD, investigational medicinal product dossier.

### Challenges in pediatric clinical translation

While all these regulatory pathways play an essential role in radiopharmaceutical development, the pediatric population presents unique challenges that make drug discovery and clinical translation particularly complex. Foremost among these is the risk of ionizing radiation with radiopharmaceuticals; this risk can be minimized but not eliminated. Children are at a higher risk of developing radiation-induced cancer when compared with adults for two primary reasons. The first is increased cell sensitivity to radiation. Although this increased sensitivity is generally accepted, there is no universal agreement on the classic linear-no-threshold (LNT) model of radiation risk (15), which assumes that any radiation dose carries some risk and does not account for alternative models, such as radiation hormesis. The lack of consensus regarding the actual risk posed by low diagnostic doses makes it difficult to accurately assess long-term outcomes in pediatric patients. Secondly, pediatric patients are actively growing, which makes their organs and tissues more radiosensitive to potential genetic damage from ionizing radiation. Furthermore, their longer post-exposure life expectancy allows more time for radiation-induces malignancies to develop (16). Combined with their lower body weight, these factors result in a higher potential risk of harm from both radiation exposure and pharmaceutical toxicity when compared with adults. Despite the overall agreement that children are more vulnerable to radiation effects, there is still no consensus on the most appropriate risk model for diagnostic-level exposures in the pediatric population (17).

Another complicating factor is the complexity of radiopharmaceutical dosimetry. While the injected dose of a radiotracer is controllable, its biodistribution can vary significantly from patient to patient and from one radiotracer to another. Even pediatric patients of similar ages and weight may exhibit markedly different biodistribution patterns for the same compound. Differences in patient height and other physiological factors can lead to variations in effective radiation does, with discrepancies as high as 18% (18). Even within single institutions using consistent dosing protocols, significant variability in effective doses can occur. For example, a study examining the commonly used bone imaging agent [^99*m*^Tc]Technetium-methylene diphosphonate ([^99*m*^Tc]Tc-MDP) in pediatric patients reported effective doses ranging from 0.7 to 1.9 mSv, despite strict controls based on weight and age (19). While these exposures are relatively low, they highlight the inherent variability in dose delivery. In positron emission tomography (PET)/computed tomography (CT) hybrid imaging, additional variability arises from the CT component. The CT dose is expressed as the CT dose index (CTDI), which is determined using phantom measurements. When these values are averaged and adjusted for the pitch factor, the result is known as the volume CT dose index (CTDI_*vol*_). When multiplied by the scan length, the Dose Length Product (DLP) is calculated, serving as a benchmark for the delivered CT dose. One study reported that the DLP for pediatric PET/CT scans ranged from 223 to 3124 mGy × cm, with corresponding PET doses varying from 2.3 to 15.9 MBq/kg (20). This high variability in CT dose stacks with variability in radiopharmaceutical dosimetry with regard to total dose. Because CT dosimetry for pediatrics has been developed through phantom scanning, small proper doses have been established, but like nuclear medicine dosimetry, the radiation dose to each individual can vary (20). Nuclear medicine imaging protocols are designed with the as-low-as-reasonably-achievable (ALARA) principle in mind to minimize radiation exposure, but the inherent variability in dosimetry makes it difficult to achieve consistent protocols in pediatric settings when compared with adult populations.

There are two major dosage guidelines in use that recommend pediatric dose activities for nuclear medicine. The primary method used in the United States is the North American Consensus Guidelines for Pediatric Administered Radiopharmaceutical Activities (NAGL), which were developed through cooperation between the Society of Nuclear Medicine and Molecular Imaging (SNMMI), the Image Gently Campaign, and the American College of Radiology in 2010 (21). NAGL guidelines were recently updated in 2024 to include a total of 29 radiopharmaceuticals used in pediatrics (22). The European Association of Nuclear Medicine (EANM) has also published an industry standard dosage card, and efforts were made to harmonize these guidelines with NAGL in 2014. The Japanese Society of Nuclear Medicine (JSNM) has published an additional high-quality dose card. Despite a general consensus on dose activities, variations exist in clinical patient dose recommendations between standards, primarily due to the way the different standards apply weight-based scaling to better established adult doses. This illustrates that even the best-established pediatric doses are derived from adult standards, rather than pediatric specific dose card values created of their own merit. In clinical practice, pediatric radiopharmaceutical doses drift even further from the established dose card guidelines. In a 2016 study (23), compliance with these dose card guidelines was shown to be as low as 27.3% for 10-year-olds being dosed with [^18^F]FDG. Radiopharmacy doses in adults are relatively standardized but they can vary widely in the pediatric population. In a recent study, the activity of pediatric doses of Technetium-99m mercapto-acetyl-triglycine ([^99*m*^Tc]Tc-MAG3) per kilogram varied between 1.85 MBq and 10.36 MBq, while the activity of Na[^123^I]I varied from 0.06 MBq to 0.22 MBq (24). This variation is partly because the NAGL and EANM provide guidelines rather than enforceable regulations. Imaging providers retain the authority to tailor protocols based on their clinical judgment and local conditions. This flexibility is often necessary because imaging equipment varies in sensitivity and resolution, forcing clinicians to balance image quality against radiation exposure. Such high variability is observed even with well-established radiotracers that have a long history of clinical use. One can only imagine the degree of variability that may exist with investigational tracers whose dosimetry is less well characterized. Investigational radiotracers are not included on the dose cards, after all.

Mindful limitation of radiation exposure is not the only concern in pediatric nuclear imaging, as several other factors contribute to its complexity. Not all children are cooperative during procedures, and in some cases, sedation or anesthesia is required, adding another layer of clinical risk. As with all pediatric interventions, there are legal and ethical considerations, and special care must be taken to ensure full transparency during the informed consent process. Due to their smaller size and different physiology, image acquisition and interpretation can also pose challenges. Pediatric organs are more difficult to image simply because of their size. Since imaging resolution is finite, there are practical limits to how well anatomical details can be resolved in smaller patients. Additionally, systems built into imaging equipment, such as certain types of attenuation correction, are typically optimized for adult patients. Physiological differences between pediatric and adult patients can also lead to significant variations in radiotracer biodistribution. In routine clinical practice, experienced radiologists are often able to recognize and interpret these differences. However, with investigational tracers, this type of interpretive expertise is not yet well established, which may further complicate image analysis in clinical trials including pediatric populations.

Another difficulty in clinical translation is subject recruitment. This is a concern across all areas of research, but it is particularly challenging in the pediatric population, given the rarity of conditions that are both severe enough to warrant pediatric radiation exposure and widespread enough to warrant specific product development. This can lead to low enrollment in clinical trials, which in turn limits the strength of the research data. There are two primary mechanisms that can rectify this. The first is the inclusion of children in adult trials. Many conditions that affect adults also affect children, and including pediatric populations in adult studies is a good way to bolster statistical strength when large pediatric cohorts are lacking. Table 2 is a representative list of current clinical trials that investigate conditions affecting both children and adults, with the potential to include mixed-age cohorts. This is the idea behind PIP-style programs, which encourage the inclusion of pediatric study data in larger trials. The second way to expand enrollment numbers is to collaborate across more jurisdictions to enroll a larger number of candidates. While the CTR in the EU and the FDA in the US have made significant progress in expanding collaboration between institutions, a broader international program would enable pediatric radiopharmaceutical data to be much more robust. Geopolitical and financial barriers remain, but increasing cooperation between international centers would significantly benefit the advancement of science.

**TABLE 2:** Representative current radiopharmaceutical clinical trials that have the potential for both pediatric and adult enrollment (25).

NCT number	Conditions	Radiopharmaceuticals	Phases	Study type
NCT00004847	Pheochromocytoma, endocrine disease, endocrine diseases	[^18^F]FDOPA, 6-[^18^F]FDA	1	Interventional
NCT00588185	Prostate cancer	[^18^F]FDG, [^18^F]Dihydrotestosterone	NA	Interventional
NCT02021604	Congenital hyperinsulinism, insulinoma	[^18^F]FDOPA	1	Interventional
NCT03541720	Neuroblastoma, pheochromocytoma	6-[^18^F]FDA	1	Interventional
NCT04205604	Congenital hyperinsulinism	[^18^F]FDOPA	2	Interventional
NCT04559217	Neuroblastoma	[^68^Ga]Ga-DOTATATE	2	Interventional
NCT04706910	Neuroblastoma, Congenital hyperinsulinism, Parkinson’s disease, Lewy body disease, neuroendocrine tumors, brain tumor	[^18^F]FDOPA	3	Interventional
NCT04847505	Neuroendocrine tumors	[^68^Ga]Ga-DOTATATE	3	Interventional
NCT04857502	Prostate carcinoma, Recurrent prostate carcinoma	[^99m^Tc]Tc-PSMA-I&S	1	Interventional
NCT04888481	Neuroendocrine tumors	[^68^Ga]Ga-HA-DOTATATE	2	Interventional
NCT04895631	Hyperparathyroidism, primary	[^18^F] Fluorocholine	3	Interventional
NCT04979611	Insulinoma	[^68^Ga]Ga-NOTA-exendin-4	1	Interventional
NCT05069220	Neuroendocrine tumor, neuroblastoma, pheochromocytoma, paraganglioma	[^18^F]MFBG, [^68^Ga]Ga-DOTATATE	1	Interventional
NCT05088798	Hyperinsulinism	[^18^F]FDOPA	2	Interventional
NCT05155280	Anorexia nervosa	[^11^C]DASB	NA	Interventional
NCT05228106	Solid cancers	[^68^Ga]Ga-PSMA-617	NA	Observational
NCT05278208	Central nervous system tumors	Lutetium Lu 177 dotatate	1, 2	Interventional
NCT05442151	Neoplasms	[^18^F]FAPI-74	NA	Interventional
NCT05553041	Glioma, high grade glioma, diffuse glioma	[^18^F]Fluciclovine	1	Interventional
NCT05555550	Low-grade glioma, malignant glioma	[^18^F]Fluciclovine	1	Interventional
NCT05632562	High grade glioma	[^18^F]FET	1	Interventional
NCT05752097	^18^F-FAPI PET/CT examination renal puncture biopsy	[^18^F]AlF-NOTA-FAPI-04	NA	Observational
NCT05826158	Neuroblastoma	[^18^F]MFBG	NA	Interventional
NCT05889312	Cancer, diagnosis, resistant cancer, response, acute phase	[^18^F]FSPG	NA	Observational
NCT06094530	Neoplasms	[^18^F]FAPI-RGD	NA	Observational
NCT06145633	Castration-resistant prostate carcinoma, Metastatic prostate adenocarcinoma, stage IVB prostate cancer AJCC v8	Gallium Ga 68 Gozetotide, Lutetium Lu 177 Vipivotide Tetraxetan, [^18^F]FDG	2	Interventional
NCT06209853	Prostatic neoplasms	DEVICE: PSMA PET/CT	NA	Observational
NCT06288113	Castration-resistant prostate carcinoma, stage IVB prostate cancer AJCC v8	Gallium Ga 68 Gozetotide, Lutetium Lu 177 Vipivotide Tetraxetan	2	Interventional
NCT06298916	Metastatic sarcoma, esophageal cancer, gastric cancer, pancreatic cancer, colorectal cancer, sarcoma	[^64^Cu]Cu-LNTH-1363S	1, 2	Interventional
NCT06474533	Intracranial neoplasm	[^18^F]FET	NA	Observational
NCT06635993	Primary aldosteronism concurrent with autonomous cortisol secretion	[^68^Ga[Ga-Pentixafor	NA	Observational
NCT06813898	Biochemically recurrent prostate carcinoma, prostate adenocarcinoma	Flotufolastat F18	1	Interventional
NCT06852807	Neuroblastoma	[^18^F]MFBG	NA	Observational
NCT06962202	Cushing syndrome		NA	Observational
NCT07064746	Neuroblastoma (NB)	[^123^I]MIBG	NA	Observational
NCT07138716	Medullary thyroid carcinoma	[^68^Ga]DOTA-CCK-FS	NA	Interventional

### Ethical considerations in pediatric nuclear medicine

While the wide-ranging applications of pediatric nuclear medicine are well recognized, the ethical landscape remains somewhat complex. The overarching principle is that pediatric patients are a vulnerable population (26), which adds an additional layer of complexity to the ethics of clinical translation. The core ethical principles relevant to the clinical translation of pediatric radiotracers include non-malfeasance, beneficence, and justice. Beneficence is always a central concern in clinical research. While patients may potentially benefit from a new treatment or diagnostic tool, randomized studies inherently make it impossible to predict which individuals will benefit due to the nature of trial design (27). Prior to a clinical trial, it is not yet known whether any specific individual will gain benefit from the intervention being tested. Thus, ethical justification relies on weighing potential risks against anticipated benefits. Non-malfeasance, the principle of “do no harm,” currently serves as the primary ethical driver in pediatric clinical research. While the commitment to avoiding harm to children is universally agreed upon, additional considerations must be addressed, particularly when ionizing radiation or investigational agents are involved.

Justice is a more complex ethical principle, involving fairness and the equitable distribution of both the risks and benefits of research. One could argue that current regulatory frameworks may unintentionally undermine justice for the pediatric population as children are significantly less likely to access investigational treatments that could potentially provide benefits. This can often result in the pediatric population being underserved for a variety of reasons, including low study recruitment numbers, especially in cases of rare diseases, as well as a disproportionately low allocation of major granting agency funds dedicated to pediatric research (28). In a recent narrative review of 35 years of meta-[^131^I]iodobenzylguanidine ([^131^I]mIBG) studies, over 1,500 pediatric patients with high-risk neuroblastoma have been enrolled worldwide. However, only one-third of patients show a reduced tumor burden, and the long-term outcomes in children remain unclear; comparative studies in pediatric populations are lacking (29). The disparities are not common in adult clinical trials. The increased liability of pediatric research also financially disincentivizes drug discovery in the pediatric population. These factors can prevent pediatric patients from receiving medical benefits from investigational treatments, which directly opposes the ethical concept of distributive justice. One attempt to rectify this ethical shortfall is the FDA’s Pediatric Research Equity Act (PREA), which requires certain drug trials to include pediatric patients. However, most radiopharmaceutical developments are typically waived or deferred under this act due to the potential safety concerns associated with radiopharmaceuticals. PREA is merely a guideline and offers little regulatory strength to force improvement.

Another key ethical consideration is informed consent. While older children may understand the potential risks and benefits, younger children typically cannot. Therefore, special care must be taken to ensure that ethical standards are upheld throughout the consent process. This is further complicated by the presence of complex guardianship or custody arrangements, which can add legal and logistical challenges. To uphold the principles of beneficence and justice, improved frameworks are needed to ensure that pediatric patients have appropriate and equitable access to investigational radiopharmaceuticals that may offer meaningful clinical values.

There is, however, a mechanism that addresses this issue on a limited scale. The FDA provides an additional regulatory pathway known as “expanded access,” also referred to as “compassionate use.” In the EU, this falls under the category of “Named Patient” or “compassionate use,” while Health Canada has a “Special Access Program.” Expanded access allows for the use of investigational products outside of clinical trials provided that strict criteria are met. The patient must have a serious or life-threatening condition for which no comparable or satisfactory alternative therapy available to diagnose, monitor, or treat the patient’s disease or conditions, and enrollment in a clinical trial must be impractical or unavailable (30). Most importantly, the potential benefits of the investigational treatment must outweigh the potential risks. This exemption requires submission of a single-patient IND application or an amendment to an existing IND to the FDA, along with institutional review board (IRB) approval. While the provisions outlined in 21 CFR 312.300 are detailed and complex, they do provide a pathway for a small number of patients to receive investigational treatment through expanded access. This pathway is not intended to serve as a regulatory loophole and demands robust rationale and justification from the treating physician, sponsor, and investigator. Although expanded access can facilitate treatment for individual patients, it is not designed to evaluate safety or efficacy across a larger population and is, therefore, less suited for product development than a traditional IND. However, it remains a valuable option for pediatric patients facing life-threatening conditions with no available alternatives. In some cases, such as with the radiopharmaceutical Lutathera, a [^1^77Lu]Lu-labeled theranostic agent targeting somatostatin receptors, data from patients treated under expanded access contributed to the eventual FDA approval of the drug (31).

## Perspectives

While several regulatory pathways exist for the clinical translation of radiopharmaceuticals, they are primarily designed for the adult patient population. Although it is understandably more ethical to conduct clinical trials in adults, certain pediatric specific pathologies differ significantly from their adult counterparts. When investigational radiopharmaceuticals reach first-in-human trials, these are typically conducted in adults. Following these trials, there is rarely any additional formal effort to evaluate the potential impact of these treatments or diagnostic tools on the pediatric population, especially in the short term. The potential liability and the challenges of risk justification pose significant barriers to pursuing pediatric applications. Exposing children to potential health complications from radiation exposure or pharmaceutical side effects is difficult to ethically justify.

However, emerging technologies may help reduce some of these risks as they become more established. One promising direction is the development of radiopharmaceuticals with improved specificity, selectivity, and deliverability (10). These characteristics allow for more targeted treatment of diseases, reducing radiation exposure to healthy tissues and in turn decreasing the overall dose required for effective imaging or therapy. Of particular interest is the field of radiotheranostics, which focuses on developing pairs of radiopharmaceuticals that target the same *in vivo* binding site: one labeled with a low energy diagnostic isotope for imaging, and the other with a high energy isotope for therapy (32). This field has seen rapid development (33), especially in the field of pediatric oncology (34), with many new tracers entering clinical trials. Despite promising preclinical and early clinical outcomes, the number of patients remains small, and more data is needed to support a broader application.

There are also advancements being made on the technical side. Increasing the sensitivity of detectors in imaging equipment allows for high quality images to be acquired with much lower radiopharmaceutical doses. One example is the whole-body PET scanner, which has been shown to produce clinically comparable images using only 1/40th of a standard dose (35). The benefits for clinical research are clear, as tracer efficacy could be evaluated at significantly lowered dose and reduced risk. Not only would the radiation burden be minimized, but any unintended pharmacological effects of the carrier compound would also be reduced. Additionally, dynamic imaging for pharmacokinetic studies could cover a much broader field of view, no longer limited to a small region at a given time. As imaging equipment continues to improve, it becomes possible to achieve lower radiation burden while still maintaining high clinical quality. A recent study (36) found that simulating a reduction in dose from 1.9 MBq/kg to 1.2 MBq/kg of [^18^F]FDG maintained an acceptable level of image quality on PET/MRI scan reconstructions. However, the image quality was significantly worse at a dose of 0.9 MBq/kg. Another emerging advancement is artificial intelligence (AI) radiomics, which applies AI to image interpretation (37). Once trained on sufficiently large datasets and refined for accuracy, these models have the potential to reduce the subjectivity inherent in radiology assessments. By minimizing human biases, such tools could help standardize data interpretation, thereby improving the overall quality and consistency of experimental results. AI tools also have the potential to improve image quality and reduce patient dose. For example, deep learning was used to denoise kinetic modeling, maintaining image quality down to as low as 1/10^th^ of a standard PET dose (38). In addition, inter-institutional collaborations and data sharing are critical to expedite the translation of radiopharmaceuticals for clinical investigation, especially when pediatric case numbers are relatively small compared to those of adult patients. Overall, improved technologies in more specific imaging agent development, imaging protocol refinement, instrument upgrades, dose optimization, harmonized regulatory and ethical safeguards can reduce risk in pediatric investigations, including radiation exposure, pharmacokinetics mismatch, ethical concerns, and off-target toxicity, which ultimately improve image quality with enhanced safety and benefit these underserved populations in clinical trials.

While there are several pathways for clinical translation, significant challenges remain in translating investigational radiopharmaceuticals for the pediatric population. Even with the most thorough preclinical validations, these drugs typically must be tested in adults before pediatric clinical trials are even considered. The current pathway to bring new radiopharmaceuticals to market remains excessively expensive and time-consuming. At the same time, novel radiopharmaceuticals have the potential to diagnose and treat illnesses that have historically been difficult to manage. A more streamlined methodology is needed to facilitate the clinical translation of these agents, especially for highly specialized radiopharmaceuticals that may lack broad commercial appeal. Since many pediatric specific diseases are rare, there is little financial incentive for large companies to develop appropriate tracers, leaving much of this research to academic institutions. Streamlining the approval process could reduce the cost of tracer development, but it must be done with care to ensure that patient safety is not compromised.

## Conclusion

The clinical translation of radiopharmaceuticals into pediatric care is full of challenges but also filled with opportunity. Current FDA rules help guide research, but they don’t always work well for pediatric cases. Ethical imperatives—safety, beneficence, and justice—must remain at the forefront, especially when dealing with vulnerable populations. Advances in radiopharmaceutical design, imaging technology, and AI analytics offer pathways to reduce radiation dose and improve targeting, thus lowering risk. However, without a dedicated framework that explicitly considers pediatric needs, these innovations may remain underutilized in clinical practice for children. To bridge this gap, coordinated efforts between regulators, researchers, industrial shareholders, and clinicians are essential. Creating pediatric-specific guidelines, expanding access to investigational agents in life-threatening conditions, and fostering early dialogue with regulatory bodies could collectively enhance the pace and safety of pediatric radiopharmaceutical development. Ultimately, achieving equity in molecular imaging requires a commitment not only to innovation but also to inclusion—ensuring that pediatric patients are not left behind in the progress of precision medicine.
